# Parental Emotional Availability and Family Functioning in Adolescent Anorexia Nervosa Subtypes

**DOI:** 10.3390/ijerph20010068

**Published:** 2022-12-21

**Authors:** Michela Criscuolo, Chiara Marchetto, Alessandra Buzzonetti, Maria Chiara Castiglioni, Lucia Cereser, Pierandrea Salvo, Valeria Zanna

**Affiliations:** 1 Anorexia Nervosa and Eating Disorder Unit, Child Neuropsychiatry, Department of Neuroscience, Bambino Gesù Childrens’ Hospital IRCCS, 00165 Rome, Italy; 2 Center for Pediatric Palliative Care, Bambino Gesù Childrens’ Hospital IRCCS, 00165 Rome, Italy; 3 Center for Eating Disorders and Weight, 30026 Portogruaro, Italy

**Keywords:** parent–child relationships, emotional availability, adolescence, family relationships, anorexia nervosa, eating disorders, restrictive anorexia nervosa, binge–purge anorexia nervosa

## Abstract

Emotional availability (EA) is a complex construct describing the emotional bond between parents and child, and it refers to support, sensitivity, warmth and closeness. Few studies have investigated the perception of parental EA and its association with dysfunctional eating pattern. The aim of the study is to explore the perception of mothers’ and fathers’ EA of adolescents with anorexia nervosa (AN) and any differences between the two subtypes of binge–purge (B/P) or restrictive (R) AN. Furthermore, it investigates the association of parental EA with AN symptomatology and with patients’ perception of family functioning, which is identified as a maintenance factor for AN. A total of 60 adolescents between 12 and 18 years and their parents (*n* = 120) were recruited in two eating disorder (ED) specialized care centers. Patients completed the LEAP and the FACES IV questionnaires evaluating parental EA and family functioning, respectively. Results showed no difference between AN subtypes, but a greater perception of mother when compared to father EA was found. Moreover, the EA construct was found not to be associated with ED symptomatology but with a greater positive family functioning. Our study is the first that explores EA in AN, and results suggest the importance of considering parents’ emotional engagement as part of the treatment core, together with the eating symptomatology management.

## 1. Introduction

Emotional availability (EA) is a complex construct describing the emotional bond between parents and child, and it refers to support, sensitivity, warmth and closeness [1].

EA assessment consists of four parental dimensions: sensitivity, which refers to the adult’s capacity to be in tune with the child, to understand and to respond adequately to their signals, to be flexible and to know how to negotiate and to resolve conflicts; structuring, which refers to the adult’s capacity to support the child’s learning and exploration, without being overstimulating, but knowing how to establish limits and rules; non-intrusiveness, which refers to the degree to which the adult leaves the right space for the child’s initiative, not replacing them; and non-hostility, which indicates the ability to have a calm and patient attitude towards the child, not devaluing or negative, and to be able to manage anger and aggression. For what concerns the child, EA is measured through the dimensions of responsiveness, the ability to feel pleasure in the relationship with the adult, being open and available and at the same time developing their own autonomy; and involvement, the ability to involve the adult and to share activities with them.

The construct was initially studied with observational methods in the context of the parent–child relationship, in particular the mother–child dyad [2], leaving the paternal figure and the subsequent stages of development in the background. Subsequently, it was noted how the existence of an emotional, flexible and welcoming relationship with the primary caregiver is fundamental in the growth of the individual [3].

Specifically, taking into consideration the age group up to 18 months of life, the study investigated the role of intergenerationality within the primary dyad: a personal history of deprivation during the childhood of the mothers had made these women less capable of making themselves emotionally available to their children. Emotional unavailability, in turn, characterized by negativity and inadequacy, was a risk factor for the onset of psychopathology in children (depressive symptoms, anxiety and behavioral problems) [4,5,6].

Lum and Phares [7] also extended the study of this construct to adolescence and early adulthood, to investigate the perception that children have of parental EA, considering both the maternal and the paternal figure. In this phase of life, EA takes on a different meaning: if during childhood, it means being physically present to welcome and satisfy the primary needs of children, in adolescence, it refers to the more emotional state of “being there”, in which the relationship becomes more equal and parents offer more mature support to their children [5,8].

It is highlighted that the construct is consistently associated with the functioning of children and their psychological well-being: several studies have observed that low levels of parental EA correspond to greater emotional and behavioral problems of children, as well as difficulties in social adaptation [4,7,9,10,11].

Furthermore, many children perceive, from infancy to adolescence, a lower parental EA [12,13] and a gender difference between parents: mothers appear to be more emotionally available, while fathers are considered less available, especially by adolescent girls [11,12,14,15,16]. However, despite a general decrease in perceived parental support from childhood to adolescence, the perception of the parent’s EA and closeness continues to play a decisive role in the emotional adaptation of children, constituting a protective factor during the adolescent period, full of turmoil and change [11,17,18]. 

Despite many studies on family relationships as a factor in the maintenance of eating disorders (ED) in the developmental age but also as a resource for a good outcome of the treatment, studies that have investigated the perception of parental EA of patients with ED are poor. Swarr and Richards [19] and Byely et al. [20] observed a positive correlation between mother–daughter/father–daughter relationships and healthy eating patterns in daughters, stating that EA plays a protective role in the etiology of ED. However, the study by Cordero and Israel [21] does not detect any correlation, even in the mother–daughter relationship, contradicting previous studies. 

In recent decades, the literature on ED maintenance factors has focused heavily on different aspects of relationships and family functioning. Despite the important methodological differences, which have often led to conflicting results, there are some elements on which the literature agrees. For example, families of patients with AN show worse general family functioning than controls [22]. In particular, studies on caregiving have noticed positive features and emotions but also negative aspects, such as high levels of expressed emotion and criticism and a worse handling of psychological distress compared to control [23]. 

Several differences were also found between patients with restrictive anorexia nervosa (AN-R) and patients with binge–purge anorexia nervosa (AN-B/P): the two subtypes differ in several behavioral and psychological characteristics, such as more impulsivity [24], higher rates of suicide attempts, more self-injurious behavior and substance use [25,26] and more severe ED psychopathology [27,28,29,30,31] of patients with AN-B/P. 

Regarding the family environment, patients with AN-B/P rate general family functioning significantly worse than patients with AN-R [22], especially in task accomplishment, communication and effective expression [32]. Some studies have found no differences between the subtypes [33,34,35,36], while other studies have found higher levels of emotional over-involvement among caregivers of patients with restrictive ED and higher levels of criticism among caregivers of patients with binge–purge symptomatology [37,38]. The perception of parental EA may be a part of that emotional context and of those relational characteristics of the family that research has shown to influence the severity of ED discomfort [39,40]. Thus, we intend to explore the perception of mothers’ and fathers’ EA of adolescents with AN and any differences between the two subtypes of ANR and AN-B/P, which the literature has shown to be different in terms of psychological, familiar and psychopathological features. We also intend to investigate if parental EA is correlated with AN symptomatology, in terms of body mass index (BMI) and scores on EDI-3 scales, and to patients’ perception of family functioning, which is identified as a maintenance factor for AN. 

## 2. Materials and Methods

### 2.1. Sample

A total of 60 adolescent patients and their parents (*n* = 120) were recruited in two Italian Centers specialized in ED care. Inclusion criteria were female sex, age between 12 and 18 years and diagnosis of AN according to the Diagnostic and Statistical Manual of Mental Disorders (5th edn, Text Revision; DSM-5) [30]. The Italian version of the Schedule for Affective Disorders and Schizophrenia for School-Age Children/Present and Lifetime Version (K-SADS-PL DSM- 5) [41] was used to corroborate psychiatric diagnoses. Patients with psychotic symptoms, a Wechsler Full Scale score < 80, significant current general medical instability and history of substance abuse were excluded. We also excluded patients with psychiatric disorders in parents and single-parent families.

All patients and parents gave their consent to participate. They were all Caucasian. The mean age of parents (all biological) was 46.7 years (SD = 4.7) for mothers and 49.8 years (SD = 5.2) for fathers. Sociocultural level was medium-high, and the majority of f amilies were united (98%) with more than one child (100%). Patients’ medium age was 14.6 years (SD = 1.4) for the AN-R group and 15.2 years (SD = 1.4) for the AN-B/P group. Considering the whole sample, the mean body mass index (BMI) was 17.2 kg/m^2^ (SD = 4.4). No significant differences were found between the two groups for BMI (t = 0.032, df = 41, *p* = 0.975). 

### 2.2. Procedures

The diagnostic evaluation protocol was performed by a multidisciplinary team and was extensively described in another study [42]. It consisted of a psychiatric consultation, an individual and family interview, an anthropometric and psychopathological evaluation and a nutritional consultation. Additionally, a battery of self-administered psychological and eating questionnaires were administered to patients, who completed them during the clinical evaluation. The study was approved by the Ethics Committees of both the structures involved.

### 2.3. Measures

The Lum Emotional Availability of Parents (LEAP) [11] was used to assess the perception of parental EA. The self-report questionnaire is divided into two versions, maternal (LEAPm) and paternal (LEAPp), each consisting of 15 items based on a six-point Likert scale (from 1 = Never to 6 = Always). The total score is given by the total of the single items, ranging from a minimum of 15 to a maximum of 90. The higher the score is, the higher the perception of EA. The Italian version has an internal validity of α = 0.90 for the maternal version and α = 0.94 for the paternal one [9].

The Italian version of the Family Adaptability and Cohesion Evaluation Scales (FACES-IV) [43] was used to evaluate family functioning. The self-report test consists of 62 items based on a Likert scale and evaluates six dimensions organized in two balanced scales (cohesion and flexibility) and four unbalanced scales (Rigid, Chaotic, Enmeshed and Disengaged). The balanced scale evaluates normal functioning and refers to the central and moderate area of the report; the four unbalanced scales are related to dysfunctional functioning and represent the extreme scores of the cohesion and flexibility scales. The strength of this version compared to the previous one is the presence of the Family Communication and Family Satisfaction scales, which have proved to be valid and reliable. For the Italian version, the reliability range is between 0.78 and 0.92 [44].

The Eating Disorder Inventory (EDI-3) [45] was used to evaluate the specific psychopathology associated with ED. The answers to the 91 items which compose the questionnaire help to elaborate a profile of the patient with ED divided into three ED-specific scales (Drive for Thinness—DT; Bulimia—B; Body Dissatisfaction—BD) and nine general psychopathological scales (Low Self-Esteem—LSE; Personal Alienation—PA; Interpersonal Insecurity—II; Interpersonal Alienation—IA; Interoceptive Deficits—ID; Emotional Dysregulation—ED; Perfectionism—P; Asceticism—A; Maturity Fears—MF). The Italian version of EDI-3 has demonstrated very good test–retest reliability, cross-informant agreement, and a good discriminating validity [46].

### 2.4. Statistical Analyses

Data are represented as number and percentages in parentheses (%). The Student’s *t*-test for independent and dependent samples was performed to compare variables between groups. Separately, the R Pearson test was used to investigate correlations among parental EA, family functioning, BMI and EDI-3 different scales’ scores. Results were significant for *p*-value <0.05. Statistical analyses were performed using IBM SPSS Statistics version 21.

## 3. Results

### 3.1. Parental EA Perceived by Patients and Differences between Groups

Regarding the whole sample of patients with AN, results indicate that mothers are perceived as more emotionally available than fathers. No differences in EA perception were found between the two groups of patients diagnosed with AN-R or AN-B/P (Table 1).

### 3.2. Parents’ EA and AN Symptomatology

No correlation between parents’ EA and AN symptomatology, measured by EDI-3, was found. A correlation trend between fathers’ EA and the Body Dissatisfaction scale was found (r = 0.289, *p* = 0.56). No correlations between EA and BMI were detected.

### 3.3. Parents’ EA and Family Functioning

A positive correlation between mothers’ and fathers’ EA perception and cohesion and flexibility scales at FACES-IV completed by patients was found (Table 2). Moreover, a positive correlation with Family Communication and Satisfaction scales was detected. Finally, a negative correlation with Disengagement and Chaotic scales was found. Raw scores of FACES-IV questionnaire completed by patients can be consulted in Supplementary Materials (Table S1).

## 4. Discussion and Conclusions

Previous studies on the population of adolescents with ED had shown conflicting results between the construct of parental EA and dysfunctional eating pattern. Our study is the first that explores EA in AN in developmental age and any differences between the subtypes of anorexia nervosa. The results did not show differences between the two groups or an association with the severity of ED symptoms, in agreement with the study by Cordero and Israel [21], which found no correlation between the construct of EA and dysfunctional eating patterns. The lack of significant relationships between daughters’ eating and paternal and maternal EA is in contrast with the few available studies which have found a correlation between these variables [19,47]. It is possible that EA may not be perceived as a problem in the acute phase of the disease, as patients may be more concerned about parental control overeating and problem behaviors. However, parental EA could play a role in the later stages of treatment and influence outcomes, where it is now known that the involvement of family members in the care pathway of AN in adolescence is a critical factor for a positive prognosis of the disease [48,49].

Regarding the whole group of patients with AN, a greater perception of maternal EA compared to paternal EA is confirmed in the adolescent group, in line with previous studies on the non-clinical population. A review study from Gale [50] investigated the role of the father–child relationship as a risk or maintenance factor of ED in developmental age. Results showed that within the relationship of the father and child, and especially with daughters, there are several themes such as conflict and communication, parental protection and psychological control, emotional regulation and self-esteem, and self-perfectionism that appear to condition the child’s level of self-determining autonomy and can consequently impact maladaptive eating attitudes and psychopathology. Authors recommended workingwith fathers to encourage free expression of ideas and foster a sense of autonomy through compromise and collaboration with their adolescent child.

Another interesting result is the association between parents’ EA and positive family functioning in terms of cohesion and flexibility. There is also a positive correlation with family communication and satisfaction. Family functioning and especially the constructs of cohesion and flexibility have been correlated with AN symptomatology, highlighting how their low presence can be a factor in the maintenance of the disease and how, consequently, it should be considered a therapeutic target. Therefore, it is desirable that subsequent studies investigate the potential interaction between EA, family functioning and AN symptoms to analyze the aspects related to the maintenance of the disorder and the factors on which the therapy should focus more. It would also be interesting to investigate whether at the end of the integrated treatment, which necessarily involves parents in this age group, the patients still perceive an imbalance between mother and father or whether they are both on the same level.

A recent study assessing post-treatment changes in family functioning among families of adolescents with severe restricting eating disorders (REDs) seems to confirm this hypothesis [51]. Through an observational instrument, the clinical Lausanne Trilogue Play (LTPc) [52], authors have observed a significant improvement in the fathers’ score and engagement at the end of integrated treatment. These findings suggest that fathers tend to be disengaged from the caregiving role, underlining the importance of encouraging a greater paternal involvement in the treatment. In line with previous studies, authors conclude that when fathers play an active role, it could improve the quality of family interactions and patient’s outcomes. In this direction, it may be useful to work from the initial stages of the treatment on several aspects—on one side to encourage parents to take on management of the meal, through the support of clinicians and in integration with hospital work, and in addition, to have them focus on the parent–child relationship, and in particular on the emotional and communicative aspects that characterize the maternal and paternal relationships separately. 

The limitations of this study are the small size of the clinical sample and the absence of a clinical or normative control group, which would have allowed a greater insight into the differences related to the construct of parental EA in the different subtypes of ED and in the non-clinical population.

## Figures and Tables

**Table 1:** EA perceived by patients and differences between groups.

	AN-R	AN-B/P	t (p)	AN	t (p)
Mothers’ EA (SD)	68.57 (20.6)	68.65 (12.4)	−0.018 (0.986)	70.44 (13)	4.7 (0.000)
Fathers’ EA (SD)	63.10 (15.9)	59.59 (17.9)	0.696 (0.490)	61.08 (16.9)	

EA, emotional availability; AN-R, restrictive anorexia nervosa; AN-B/P, binge–purge anorexia nervosa; AN, anorexia nervosa.

**Table 2:** Emotional availability perceived by patients and family functioning.

	Cohesion	Flexibility	Disengaged	Enmeshed	Rigid	Chaotic	Communication	Satisfaction
Mothers’ EA	0.516 **	0.481 **	−0.500 **	0.064	0.132	−0.309 *	0.398 **	0.394 **
Fathers’ EA	0.674 **	0.619 **	−0.632 **	0.143	0.114	−0.275	0.579 **	0.472 **

EA, emotional availability; ** significant correlation at 0.01 level (2-code); * significant correlation at 0.05 level (2-code).

## Data Availability

Not applicable.

## Supplementary Materials

The following supporting information can be downloaded at: https://www.mdpi.com/article/10.3390/ijerph20010068/s1, Table S1: Raw scores of FACES-IV scales.
