# Supplementary material for: Parental Emotional Availability and Family Functioning in Adolescent Anorexia Nervosa Subtypes
Source: Int J Environ Res Public Health. 2022 Dec 21;20(1):68. doi: 10.3390/ijerph20010068 (PMC9819780; doi:10.3390/ijerph20010068)
Supplement: Supplementary file 1 [file ijerph-20-00068-s001.zip › ijerph-1975133-supplementary.pdf]

Table S1. Raw scores of FACES-IV scales

	Cohesion (SD)	Flexibility (SD)	Disengaged (SD)	Enmeshed (SD)	Rigid (SD)	Chaotic (SD)	Communication (SD)	Satisfaction (SD)
AN-R	26.9 (4.8)	25.1 (4.4)	15 (3.9)	14.4 (5.3)	19.1 (4)	16.8 (4.5)	35 (7.6)	45 (22.7)
AN-B/P	25.7 (5.3)	23.7 (5.4)	17 (5.1)	15.6 (4.1)	18.5 (4)	18.5 (4)	33.9 (7.6)	43.2 (25.4)

AN-R, restrictive anorexia nervosa; AN-B/P, binge-purge anorexia nervosa
